# Sustained Release of Bone Morphogenetic Protein-2 through Alginate Microbeads Enhances Bone Regeneration in Rabbit Tibial Metaphyseal Defect Model

**DOI:** 10.3390/ma14102600

**Published:** 2021-05-17

**Authors:** Junhyung Kim, Seoyun Lee, Yonghyun Choi, Jonghoon Choi, Byung-Jae Kang

**Affiliations:** 1 College of Veterinary Medicine and Institute of Veterinary Science, Kangwon National University, Chuncheon 24341, Korea; perfectnk@nate.com; 2 Department of Veterinary Clinical Sciences, College of Veterinary Medicine and Research Institute for Veterinary Science, Seoul National University, Seoul 08826, Korea; suyoon92@snu.ac.kr; 3 BK21 FOUR Future Veterinary Medicine Leading Education and Research Center, Seoul National University, Seoul 08826, Korea; 4 School of Integrative Engineering, Chung-Ang University, Seoul 06974, Korea; dydgus5057@gmail.com (Y.C.); nanomed@cau.ac.kr (J.C.)

**Keywords:** bone morphogenetic protein-2, alginate microbeads, bone regeneration, sustained release, controlled release

## Abstract

Bone morphogenetic protein-2 (BMP-2) is widely used to enhance bone regeneration. However, because of its short half-life and rapid disappearance, large amounts of BMP-2 are needed, leading to unintended side effects. In this study, BMP-2-encapsulated alginate microbeads (AM) were used to enhance bone regeneration. Enzyme-linked immunosorbent assay confirmed the sustained release of BMP-2 from AM. Vascular endothelial growth factor (VEGF)-adsorbing aptamer-conjugated hydroxyapatite (Apt-HA) was used for osteoconduction and dual delivery of VEGF and BMP-2. For in vivo bone regeneration evaluation, the grafts (1) Apt-HA + phosphate-buffered saline (PBS), (2) Apt-HA + AM without BMP-2, (3) Apt-HA + BMP-2, and (4) Apt-HA + AM encapsulated with BMP-2 were implanted into rabbit tibial metaphyseal defects. After four weeks, micro-computed tomography (CT), histological, and histomorphometric analyses were performed to evaluate bone regeneration. The Apt-HA + AM with BMP-2 group revealed a significantly higher new bone volume and bone volume/total volume (BV/TV) in both cortical and trabecular bone than the others. Furthermore, as evaluated by histomorphometric analysis, BMP-2 AM exhibited a significantly higher bone formation area than the others, indicating that AM could be used to efficiently deliver BMP-2 through sustained release. Moreover, the combined application of BMP-2-encapsulated Apt-HA + AM may effectively promote bone regeneration.

## 1. Introduction

Bone injury and loss can be caused by trauma, surgery, infection, cancer, and degenerative diseases [1,2]. Many patients suffering from bone defects and bone loss require bone grafts or bone substitutes. Autologous bone graft is the gold standard, having all the characteristics of osteogenesis, osteoconduction, and osteoinduction. However, it has some disadvantages, such as insufficient donor tissue, multiple surgeries, and defects in the sampling area [3,4,5]. Besides, allogeneic bone grafts and xenografts have side effects due to pathogen transmission, infection, and immune responses. Thus, bone regeneration engineering has emerged as a potential new strategy to overcome these shortcomings.

Recently, regenerative medicine approaches mimicking or regulating natural bone healing processes are emerging new strategies to promote bone regeneration [6]. The regulation of the bone regeneration process involves using bone resorption inhibitors such as bisphosphonates [7] or various growth factors such as bone morphogenetic protein-2 (BMP-2), BMP-7, vascular endothelial growth factor (VEGF), and fibroblast growth factor-2. Among those options, the application of multiple growth factors mimicking the natural healing process can be used as a biomimetic drug delivery strategy to maximize osteogenic outcome and therapeutic effect. Hence, for instance, the combined application of VEGF as an angiogenic factor and BMP-2 as an osteogenic factor in bone tissue engineering may be more effective than applying a single growth factor. In this study, the bivalent aptamer-conjugated hydroxyapatite (Apt-HA), which specifically adsorbs VEGF [8], is used for synergistic bone regeneration and osteoconduction purposes.

BMPs belong to the transforming growth factor-beta superfamily, whose members strongly induce bone regeneration. Among them, BMP-2 is the most widely used for this purpose, playing a role in osteoinduction by differentiating mesenchymal stem cells (MSCs) into osteoblasts. Although BMP-2 has been a clinical alternative to autograft bone, it quickly loses its biological activity in vivo because of its short half-life, averaging 7–16 min intravenously, and rapid clearance through diffusion [9]. Due to these limitations, a supraphysiologic amount of BMP-2 is clinically required, which leads to harmful side effects, such as ectopic bone formation, edema, immune responses, soft tissue inflammation [10], and bone void formation [11]. To overcome these disadvantages, developing a delivery carrier capable of locally releasing BMP-2, with an inadequate concentration, and for a long time is needed. Therefore, various delivery carriers for BMP-2, such as collagen sponges, microbeads, nanoparticles, fibers, ceramics, and gels, are constantly being developed in a large number of preclinical studies [7,12].

Alginate, extracted from brown algae, is a natural polymer composed of α-L-guluronic acid (G) and β-D-mannuronic acid (M) chains. Sodium alginate has been investigated and is commonly used in the biomedical field because of its biocompatibility, low toxicity, and relatively low cost [13]. Alginate microbeads (AM) are multiparticle delivery systems that can provide increased drug stability and target-specific implantation. Thus, the encapsulation of AM with BMP-2 could amplify bone healing while minimizing the side effects mentioned above. Furthermore, AM fabrication for controlled release occurs under very mild conditions, excluding high temperatures or chemical cross-linking reagents [14]. In our previous research, we fabricated AM containing BMP-2 and confirmed that the sustained release of BMP-2 through AM improved bone formation in rat calvarial bone defects [15]. In the present study, we validated the efficacy of AM as a BMP-2 delivery carrier to verify the regeneration capability in the cortical and medullary bone region, refine the manufacturing method of AM, and measure the entrapment efficiency of BMP-2.

In this study, we aimed to investigate the effect of the sustained release of BMP-2 on bone regeneration by applying AM encapsulated with BMP-2 in a rabbit tibial metaphyseal model. Additionally, we evaluated the release kinetics and encapsulation efficiency (EE) of AM containing BMP-2 and hypothesized that dual delivery using both BMP-2 and VEGF would show a synergistic effect on bone healing.

## 2. Materials and Methods

### 2.1. Preparation of Alginate Microbeads Loaded with BMP-2

**Figure 1:**
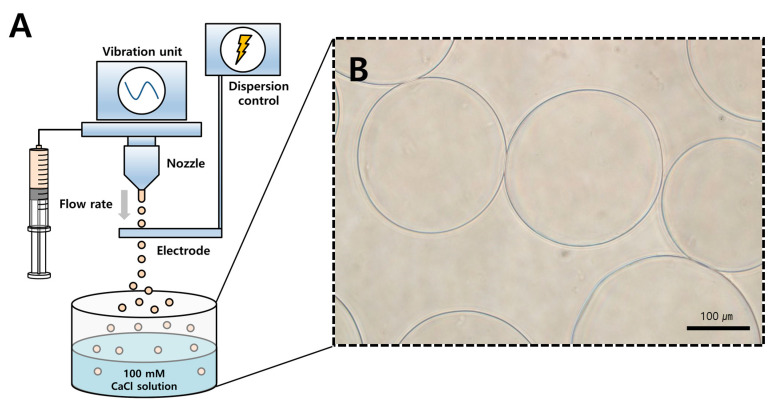
Schematic illustration of fabrication procedures and morphology of alginate microbeads (AM). (**A**) Preparation of bone morphogenetic protein-2 (BMP-2)-encapsulated AM using a vibrational nozzle technique in this study. The following parameters were used: nozzle size, 120 μm; frequency, 1000 Hz; flow rate, 23 mL/min; and electrode potential, 1000 V. (**B**) Morphology of AM encapsulated with BMP-2 in light-field microscopy. Scale bar = 100 μm.

BMP-2-encapsulated AM were fabricated following Lee et al. [15]: A solution of 1.8% (*w*/*v*) sodium alginate from Büchi Labortechnik AG (Flawil, Switzerland) was diluted to 1.2% (*w/v*) by adding distilled water. Next, BMP-2 (Novosis, CGBio, Gyeonggi-do, Korea) was added to the 1.2% (*w/v*) sodium alginate solution at a protein/polymer ratio of 3.47 μg/mg. BMP-2-encapsulated AM were produced by a vibrational nozzle technique through a Büchi Encapsulator B-395 pro (Dottikon, Switzerland) under the following optimized parameters [16]: 23-gauge needle; 120 μm nozzle size; 1000 Hz frequency; 23 mL/min flow rate; and 1000 V electrode potential (Figure 1A). The mixture droplets of alginate and BMP-2 were gelled in a 250 mL 100 mM calcium chloride solution for 30 min. Finally, BMP-2-encapsulated AM were purified through a 40 m cell strainer and twice washed with phosphate-buffered saline (PBS).

### 2.2. Morphology and Encapsulation Efficiency

The morphology of BMP-2-encapsulated AM was photographed using light microscopy (Figure 1B). To evaluate the BMP-2 retention, the amount of encapsulating BMP-2 was directly determined by measuring the total amount of BMP-2 and that of BMP-2 in AM. AM were mixed with 50 mM disodium ethylenediaminetetraacetate dihydrate (EDTA) solution for 5 min to dissolve the capsule and obtain the BMP-2. The amount of BMP-2 in AM was assayed using the rhBMP-2 enzyme-linked immunosorbent assay (ELISA) kit (R&D system Inc., Minneapolis, MN) according to the manufacturer’s instructions. The EE was determined using the following equation:

$$\mathrm{EE}\;\left(\%\right)=\frac{\mathrm{Amount}\;\mathrm{of}\;\mathrm{BMP}-2\;\mathrm{in}\;\mathrm{alginate}\;\mathrm{microbeads}}{\mathrm{Total}\;\mathrm{amount}\;\mathrm{of}\;\mathrm{BMP}-2}\times 100$$

### 2.3. In Vitro BMP-2 Release Kinetics from Alginate Microbeads

To assess the BMP-2 release kinetics from the microbeads, AM encapsulated with BMP-2 containing 6 μg of BMP-2 were placed in 2 mL PBS and incubated for 28 days at 37 °C with 80 rpm shaking. At the following time points, the supernatant was collected and replaced with a fresh PBS solution: 1, 3, 5, 7, 14, 21, and 28 days. All collected supernatant samples were stored at 70 °C. A BMP-2 ELISA kit was used to quantify the amount of BMP-2 emitted into the supernatant, as per the manufacturer’s instructions. The cumulative release of BMP-2 was also assessed.

### 2.4. Experimental Animals

In this study, a total of 12 New Zealand White male rabbits, weighing 2.5 to 3.0 kg and aged approximately 16 weeks, were used. The study was approved by the Kangwon National University Animal Experimental Ethics Committee (KW-190103-9). 

### 2.5. Rabbit Tibial Metaphyseal Defect Model for Bone Regeneration

**Figure 2:**
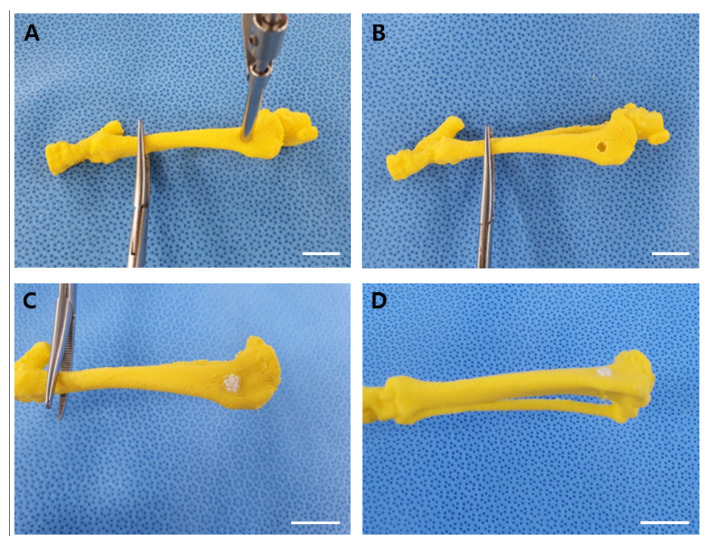
Surgical rehearsal using a three-dimensional printed bone model. (**A**) A defect was made in a three-dimensional printed tibial bone model using the drilling technique. (**B**) Three-dimensional printed bone model with proximal tibia metaphysis defect. (**C**) Lateral view with aptamer-conjugated hydroxyapatite (Apt-HA) filled in the defect. (**D**) Craniocaudal view with Apt-HA filled in the defect. Scale bars = 10 mm.

Prior to the surgery on the rabbits, surgical rehearsal was performed to minimize errors and ensure accurate surgery (Figure 2). AM encapsulated with BMP-2 containing 6 μg of BMP-2 were used for the surgery on the rabbit tibial metaphyseal defect model. Apt-HA was prepared and provided by the School of Integrative Engineering, Chung-Ang University, Seoul, Korea. For implantation, the 12 rabbits were randomly assigned to one of four experimental groups: (1) Apt-HA + PBS, (2) Apt-HA + AM without BMP-2, (3) Apt-HA + BMP-2, and (4) Apt-HA + AM with BMP-2.

Before surgery, all rabbits were acclimatized for one week. To calculate the proper drug dosage, all rabbits were preoperatively weighed. Sedation was achieved by injection of xylazine (5 mg/kg, IM). Anesthesia was induced with alfaxalone (4 mg/kg, IM) and maintained with 2% isoflurane inhalation. Tramadol (4 mg/kg, SC) was injected as an analgesic and cefazolin (22 mg/kg, SC) was injected for prophylactic antibiotics. After disinfection with alcohol-based hexidine, a 4-cm skin and subcutaneous incision was made medially in the proximal tibia. Following the incision, the periosteum was dissected as well. Using a drill bit of 3.5 mm diameter and stop device, two defect holes were drilled in the medial side of proximal tibial metaphysis, resulting in two 3.5 mm diameter and 7 mm depth defects. The distance between the two defects was adjusted to 10 mm. After rinsing with 0.9% saline, the volume of the defect was equally filled with Apt-HA in all groups. Subsequently, the defect site was filled with additional material as per their respective experimental groups. After implantation, the periosteum and skin incision site were closed with an absorbable suture material (3-0, SURGIFIT, AILEE CO., Busan, Korea) with a simple continuous technique and a surgical stapler. To provide analgesia and reduce infection risk, all rabbits were given tramadol (4 mg/kg) and cefazolin (22 mg/kg) injections after surgery. Four weeks after surgery, all rabbits were euthanized using a T61® solution, a combination of embutramide, mebenzoniumjodide, and tetracaine HCl (Intervet, Mechelen, Belgium). Their proximal tibias were harvested for micro-computed tomography (CT) and histological analyses.

### 2.6. Radiographic Assessment

Radiographic analysis was performed based on the lateral view of the tibia every two weeks. All radiographs were acquired under the same exposure conditions, with 45 kVp and 25 mAs. The radiographic images of the tibia, immediately post-surgery and 2 and 4 weeks post-surgery, were obtained. Bone regeneration was subjectively assessed through these radiographs.

### 2.7. Micro-Computed Tomography Analysis

The harvested proximal tibias were scanned along the anterior–posterior, cross-sectional, and lateral planes to evaluate bone regeneration. Micro-CT images were obtained from a SkyScan 1173 system (Bruker-CT, Kontich, Belgium). The scanning conditions were as follows: 130 kV voltage and 60 µA current. To obtain section reconstruction images, the SkyScan Nrecon software (ver. 1.6.9.8, Bruker-CT) was used. The cortical and trabecular bone regions were analyzed using the CT Analyzer software (ver. 1.14.4.1, Bruker-CT). DataViewer (ver. 1.5.1.2, Bruker-CT) was used to align the images, and the regions of interest were determined to include the defect sites. The data on new bone volume and new bone volume/total volume (BV/TV) were obtained for quantitative evaluation.

### 2.8. Histological and Histomorphometric Analyses

The harvested samples were fixed in 10% neutral buffered formalin (Sigma Aldrich, Darmstadt, Germany) for one week and decalcified in 10% (*w/v*) EDTA solution for two weeks. After dehydration, the extracted tibias were embedded in paraffin and cut into 4 µm thick sections at the central part of the defects. The sections were then stained with hematoxylin and eosin (H&E) and Masson’s trichrome to assess bone regeneration. The histomorphometric evaluation was performed in two parts: one for the cortical bone region and one for the trabecular bone region. The new bone formation area was calculated by measuring the area of the newly formed bone from the edge of the defect. The following formula was used to calculate the total defect area of the cortical bone portion: 3.5 mm × the cortical bone height (approximately 2.0 mm). In addition, the total defect area of the trabecular region was determined using the following formula: 3.5 mm × (7–the cortical bone height = approximately 5.0 mm). The area thus obtained was applied as (new bone area/total defect area) × 100, to determine the newly formed bone percentage in the cortical and trabecular bone regions.

### 2.9. Statistical Analysis

All quantitative data are expressed as the mean ± standard deviation. Additionally, all statistical analyses were performed using the GraphPad Prism version 7.0 (GraphPad Software Inc., San Diego, CA, USA). For the micro-CT and histological analyses, the Kruskal–Wallis one-way ANOVA test was performed to identify the statistical significance. Additionally, the Mann–Whitney U-test was performed to evaluate the differences between the two groups. Differences were considered statistically significant at *p*-value < 0.05.

## 3. Results

### 3.1. Morphology and EE

Under the conditions described above, BMP-2-encapsulated AM were spherical and had a diameter of approximately 250 μm (Figure 1B). The EE range of 55.76–64.42% was confirmed using the previously described method. The mean EE was 59.6%.

### 3.2. Controlled Release of BMP-2

**Figure 3:**
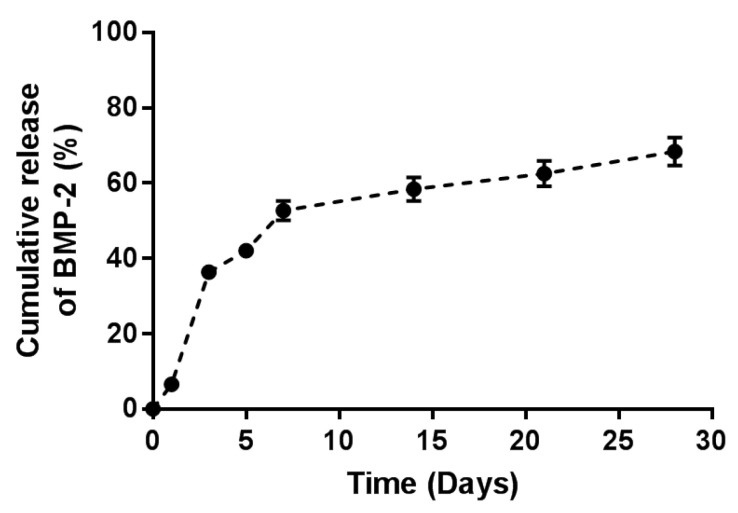
Percentage cumulative bone morphogenetic protein-2 (BMP-2) release profile from alginate microbeads encapsulated with BMP-2.

The in vitro release profile of BMP-2 from AM was evaluated as the cumulative percentage release of BMP-2 by ELISA (Figure 3). For the first 7 days, AM encapsulated with BMP-2 revealed a burst release of BMP-2. Afterward, BMP-2 had been emitted from AM continuously for up to 28 days. Approximately 50% of BMP-2 was released throughout the first 7 days. Subsequently, nearly 65% cumulative release was achieved over 28 days.

### 3.3. Radiographic Finding and Evaluation

**Figure 4:**
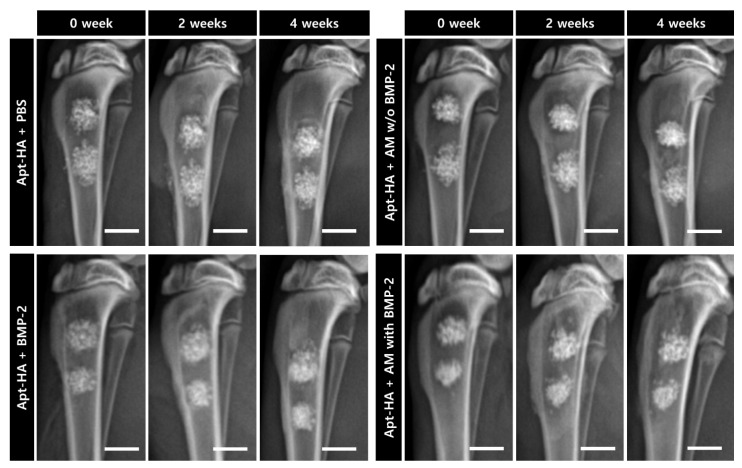
Lateral radiographs of the tibia immediately after the operation and after 2 and 4 weeks. The top left panel represents periodic radiographs of the aptamer-conjugated hydroxyapatite (Apt-HA) + phosphate-buffered saline group, the top right panel represents periodic radiographs of the Apt-HA + AM without bone morphogenetic protein-2 (BMP-2) group, the bottom left panel represents periodic radiographs of the Apt-HA + BMP-2 group, and the bottom right panel represents periodic radiographs of the Apt-HA + AM with BMP-2 group. Scale bars = 5 mm.

Radiographs were immediately acquired after surgery, as well as after 2 and 4 weeks. Periodic radiographs until four weeks did not show differences between the groups (Figure 4). In all groups, it was confirmed that the material in the defect had not yet decomposed. Moreover, radio-opacity increased with time. In the group where BMP-2 was added, it was found that the boundary of the material implanted in the defect tended to blur over time. However, this was a subjective evaluation and not an objective group comparison.

### 3.4. Micro-CT Analysis

**Figure 5:**
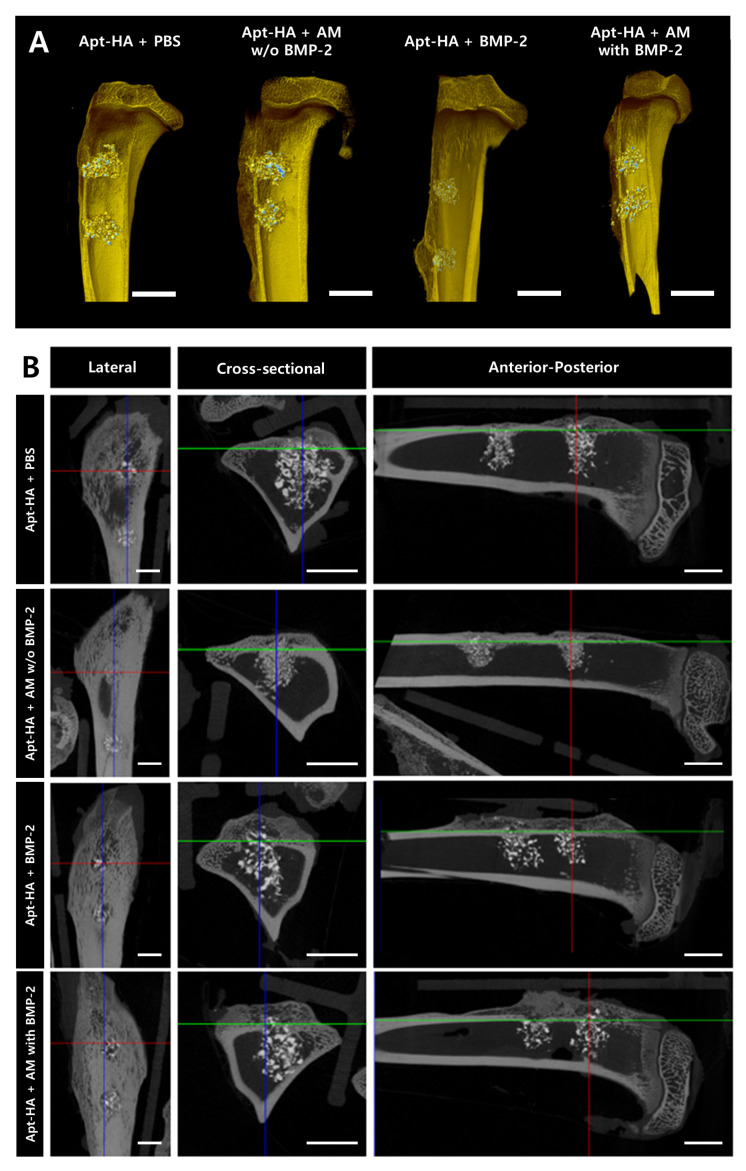
Qualitative analysis of the bone formation in the rabbit tibial metaphyseal defect. (**A**) Three-dimensional micro-computed tomography (CT) rendered reconstruction image. (**B**) Micro-CT images of the tibia defect site, including lateral, cross-sectional, and anterior–posterior views. Scale bars = 5 mm.

Three-dimensional reconstruction images of the tibia were acquired using micro-CT analysis (Figure 5A). Although the defects in all groups were filled with regenerated bone and graft materials, the differences in bone regeneration were not grossly comparable between all groups.

In addition, the lateral, cross-sectional, and anterior–posterior views of tibias of each group were obtained through micro-CT analysis. Particularly, when compared in the cross-sectional and anterior–posterior views, it was confirmed that the group containing BMP-2 showed more bone regeneration in the cortical bone area than the group without BMP-2 (Figure 5B). In the medullary bone area, estimating the dissimilarity in bone regeneration was difficult since the graft material at the defect site had not yet degraded in all groups. 

Quantitative analysis was conducted for objective evaluation. New bone volume and BV/TV were measured in the cortical and medullary bone areas, respectively.

**Figure 6:**
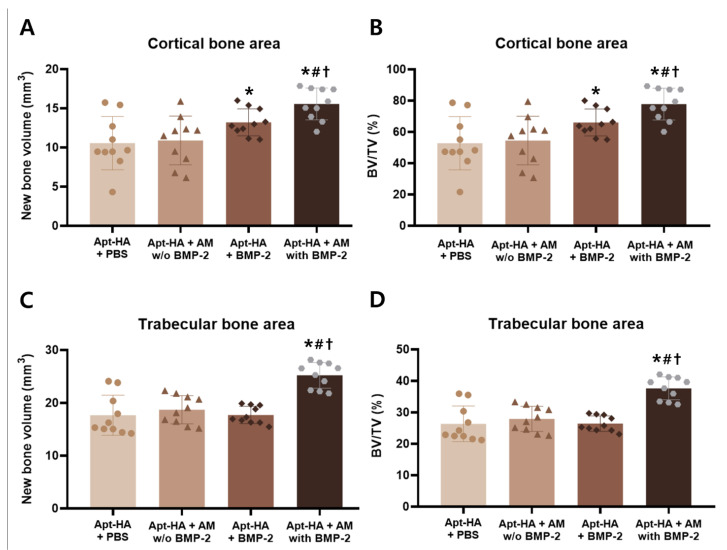
Quantitative micro-computed tomography analysis of tibia bone formation. (**A**) Quantitative analysis of new bone volume in the cortical bone area. (**B**) Quantitative analysis of new bone volume in the trabecular bone area. (**C**) Quantitative analysis of bone volume/total volume (BV/TV) in the cortical bone area. (**D**) Quantitative analysis of BV/TV in the trabecular bone area. *, #, †: *p* < 0.05 compared with aptamer-conjugated hydroxyapatite (Apt-HA) + phosphate-buffered saline group, Apt-HA + AM without bone morphogenetic protein-2 (BMP-2) group, and Apt-HA + BMP-2 group, respectively.

In the cortical bone area, the Apt-HA + AM with BMP-2 group revealed a significantly higher new bone volume than other groups (Figure 6A). Furthermore, the Apt-HA + AM with BMP-2 group showed substantially higher BV/TV values in the cortical bone area compared with the other groups (Figure 6B). In the cortical bone area, the Apt-HA + BMP-2 group had significantly higher new bone volume and BV/TV than the Apt-HA + PBS group. There was no statistical significance between the Apt-HA + PBS and Apt-HA + AM without BMP-2 groups.

In the trabecular bone area, the Apt-HA + AM with BMP-2 group had a significantly higher new bone volume and BV/TV than the other groups (Figure 6C,D). Moreover, there was no statistical significance between the three groups, except for the Apt-HA + AM with BMP-2 group.

### 3.5. Histological and Histomorphometric Analyses

**Figure 7:**
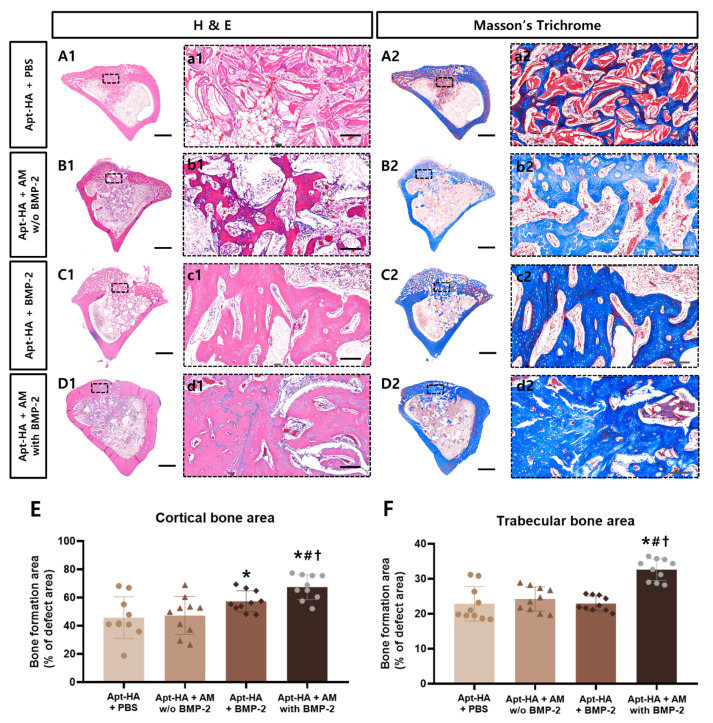
Histological and histomorphometric analysis of the defect sites at 4 weeks. Hematoxylin and eosin staining: photomicrographs of (**A1**–**D1**), and (**a1**–**d1**); Masson’s trichrome-stained sections at the center of the defect site: photomicrographs of (**A2**–**D2**), and (**a2**–**d2**). Scale bars = 2000 µm, 200 µm (zoomed-in images of the dotted rectangle). (**E**,**F**) Histomorphometric analysis to assess the newly regenerated bone at the cortical and trabecular bone areas. *, #, †: *p* < 0.05 compared with aptamer-conjugated hydroxyapatite (Apt-HA) + phosphate-buffered saline group, Apt-HA + AM without bone morphogenetic protein-2 (BMP-2) group, and Apt-HA + BMP-2 group, respectively.

For histological and histomorphometric analyses, the newly regenerated bone in the tibia defects was evaluated by H&E and Masson’s trichrome staining. All groups exhibited bony bridging in the cortical bone (Figure 7A–D).

In the cortical bone, enhanced bone formation was observed in the groups to which BMP-2 was added (Apt-HA + BMP-2 and Apt-HA + AM with BMP-2) compared with the other groups (Apt-HA + PBS and Apt-HA + AM without BMP-2). It was also confirmed that the implanted materials were still present in the defect in all groups.

Additionally, the histomorphometric analysis demonstrated that the bone regeneration of Apt-HA + AM with BMP-2 was substantially higher than that of other graft material groups in the cortical and trabecular bone areas (Figure 7E,F).

## 4. Discussion

The effectiveness of BMPs on bone formation has been reported in several previous studies. Furthermore, among all BMPs, BMP-2 is reported to have substantial and significant biological activity [17]. However, BMP-2 has a short half-life in the body. Besides, because of rapid loss through diffusion, a large amount of BMP-2 is required to obtain the desired bone regeneration [18,19,20]. Therefore, delivery carriers manufactured by the natural polymers, synthetic polymers, and proteins capable of local delivery and sustained release are constantly being developed [21]. Sodium alginate, which is one of the natural polysaccharides, has the advantages of high biocompatibility, good biodegradability, relatively low toxicity, low cost, and ease of handling [16]. In addition, alginates can be cross-linked by extruding the alginate/drug solution as microbead droplets into calcium chloride solution. Therefore, in this study, BMP-2-encapsulated AM were fabricated using an external gelling method to maintain sustained release kinetics.

The release profile of BMP-2 from AM in this study presents the typical initial burst release and subsequent sustained release patterns. When microbeads are clinically applied, the early burst release of BMP-2 can increase the recruitment of MSCs, and the subsequent sustained release can induce MSCs to differentiate into osteoprogenitor cells [15,22]. Thus, it has been demonstrated that AM can be an effective strategy for bone regeneration. Besides, the release profile graph exhibits a lower initial burst release and a longer sustained release of BMP-2 than those of our previous findings [15]. This is because, in the present study, the alginate solution was diluted with distilled water during the fabrication of microbeads instead of using 0.9% NaCl. Since sodium ions are monovalent cations, they can interfere with the intermolecular cross-linking between the divalent calcium ions and the guluronate blocks of alginate, which control the strength of the microbeads and the release of BMP-2. Then, the sodium ions in NaCl can result in reduced functional junctions and weak gelation [23]. Therefore, the use of distilled water may increase the strength of the AM and provide a stable release of BMP-2, which was in fact demonstrated through the release profile obtained using ELISA.

Although the fabrication of calcium AM is fast and simple, there could be a loss of the drug during the manufacturing process [24]. In this experiment, the directly measured EE of BMP-2 was found to be 59.6%, which is a relatively moderate efficiency with the reported range of 25–80% in various BMP-2 microsphere carriers [25,26,27]. The reduction in loading efficiency may be attributed to BMP-2 leakage into the calcium chloride solution due to a highly porous and low cross-linking matrix [28]. Generally, several factors can influence the EE: polymer and cross-linking agent concentrations, gelation time, and gelation temperature [29]. For example, in a calcium chloride solution, a higher alginate concentration can make a rapid connection with calcium ions and increase the droplet viscosity, which in turn induces low porosity and delays the drug diffusion. Thus, determining the optimal parameters for improving the EE of the BMP-2 AM in future studies is necessary.

The rabbit tibial metaphyseal defect model was used to assess in vivo bone regeneration by the sustained release of BMP-2. Unlike the calvarial defect model, the tibial metaphysis provides a suitable bony environment for evaluating cortical and trabecular bone regeneration [30]. Since the cortical defects were recovered to some extent during the experiment in all groups, a quantitative measurement of new bone volume was performed using micro-CT. A significant increase in cortical bone volume was confirmed for both groups applying BMP-2 compared with the PBS-applied control group. In the trabecular area, however, the groups with directly implanted BMP-2 showed no significant difference compared with the groups without BMP-2. Hence, it can be inferred that BMP-2 was quickly lost through diffusion because the space in the medulla in trabecular bone is larger than that in the cortical bone. These data indicate that although BMP-2 itself has an osteoinductive ability without a carrier, the sustained release of BMP-2 from AM leads to more effective bone regeneration. In terms of bone strength, in the BMP-2 AM group, bone volume fractions of both cortical and trabecular bone area were found to show significantly high BV/TV. Clinically, BMP-2-induced periosteal bone formation can expand overall long bone diameter and can reduce fracture risk by increasing the moment of inertia [31]. Moreover, the improved volume fraction of the trabecular bone contributes to the connection of bone and a reduction in porosity, which can predict the strengthened bone [32]. As a result, this finding suggests that prolonged BMP-2 release has a beneficial impact on bone functional aspects.

Another objective of the study was to indirectly evaluate the bone regeneration effects of a combination of VEGF-adsorbed Apt-HA and AM with BMP-2. In groups in which both Apt-HA and BMP-2 were applied, the rate of cortical bone regeneration was significantly higher than those with Apt-HA alone. VEGF promotes angiogenesis, enhances vascular permeability, and plays a vital role in recruiting MSCs [33,34]. In addition, its simultaneous application with BMP-2 is known to increase cell survival, cartilage formation, and mineralized bone formation [35,36]. In addition, both angiogenesis around the osteoblasts and signaling molecules, such as BMP-2, help osteoblast differentiation [37,38,39]. Therefore, by simultaneously utilizing Apt-HA and AM in this experiment, a dual delivery strategy was applied to deliver both VEGF and BMP-2, which was expected to be effective for bone regeneration. However, although Apt-HA + AM with BMP-2 may be effective for bone regeneration, further study is needed to analyze the ratio of VEGF and BMP-2 and show sequential release patterns. In addition, although no cytotoxicity was present in previous individual applications [8,15], cytotoxicity in concurrent applications should also be assessed.

## 5. Conclusions

In the present experiment, significant bone regeneration was confirmed by simultaneously applying Apt-HA with angiogenic capacity and osteoconductivity and AM capable of the sustained release of BMP-2 with osteoinduction capacity. Thus, although the BMP-2 delivery system of Apt-HA + AM encapsulated with BMP-2 has no characteristics of osteogenesis, it is expected to be a viable method to replace autogenous bone grafts using the mechanism underlying osteoconduction, osteoinduction, and angiogenesis. In conclusion, the simultaneous application of Apt-HA and AM encapsulated with BMP-2 may be a new alternative therapy for bone regeneration in patients with bone defects.

## Data Availability

The data presented in this study are available from the corresponding author upon reasonable request.
